# The COVID-19 vaccination hesitancy among Chinese individuals with diabetes and the impact on glycemic control of vaccination: a questionnaire study

**DOI:** 10.1186/s12902-022-01201-5

**Published:** 2022-12-23

**Authors:** Difei Lu, Ying Gao, Xiaojing Qi, Ang Li, Junqing Zhang

**Affiliations:** 1 Department of Endocrinology, Peking University First Hospital, Beijing, China; 2 iHealth Labs China Co.,Ltd, Beijing, China

**Keywords:** Diabetes mellitus, SARS-CoV-2 vaccination, Vaccine hesitancy, Glycemic control

## Abstract

**Objective:**

The study aimed to investigate the attitudes of people with diabetes mellitus (DM) on COVID-19 vaccination and its influence on the glycemic control.

**Methods:**

Data were collected from a consecutive series of adults (age > 18 years) with type 2 diabetes under regular follow-ups in the Integrated Care Diabetes Outpatient Clinic of Peking University First Hospital from December 1^st^ to December 31^st^ 2021. An online interview questionnaire was conducted, and demographic data including age, sex category, history of drug allergy, history of hypertension, the duration of diabetes, reasons for vaccine hesitancy (VH) and adverse reactions after each injection of vaccines was collected. Glucose levels were collected from medical records.

**Results:**

Thirty-nine (22.9%) subjects experienced VH and 131 (77.1%) people living with diabetes received inactivated vaccine against COVID-19. Hesitant individuals had a higher proportion of female gender (vaccinated group vs. VH group, 62/131 vs. 26/39, *p* = 0.044), higher baseline glycosylated hemoglobin A_1c_ (HbA_1c_) (vaccinated group vs. VH group, 6.56 ± 0.95% vs. 7.54 ± 2.01%, *p* < 0.001) and elevated baseline postprandial blood glucose (PBG) (vaccinated group vs. VH group, 8.32 ± 1.97 mmol/L vs. 9.44 ± 2.94 mmol/L, *p* = 0.015). Subjects of male gender (*p* = 0.025) and history of hypertension (*p* = 0.021) were likely to get vaccinated, while higher HbA1c was negatively associated with an elevated propensity to receive anti-COVID-19 vaccine (*p* = 0.003). Most common reasons for hesitating to receive COVID-19 vaccination were worrying about the possibility of leading to other diseases (30.8%), followed by fearing of glucose variation (17.9%). Systemic adverse reactions were reported in 30.5% individuals after the first injection of inactivated vaccines, and resolved within 3 days in medium. Fasting blood glucose (FBG) decreased significantly after the third injection compared with FBG after the second dose (second vs. third, 6.78 ± 1.24 mmol/L vs. 6.41 ± 1.30 mmol/L, *p* = 0.027). HbA_1c_ reduced significantly from 6.56% before vaccination to 6.35% after the second injection (*p* = 0.012).

**Conclusions:**

Our study demonstrated that vaccine hesitancy was lower among male subjects and people with hypertension, while vaccine confidence was reduced in people with poor glycemic control. HbA_1c_ level was lower along with vaccination.

## Introduction

The Coronavirus Disease 19 (COVID-19) caused by the Severe Acute Respiratory Syndrome- Coronavirus 2 (SARS-CoV-2) has resulted in a worldwide pandemic and a dramatic impact on world’s health care system and economy since the beginning of 2020. People with diabetes mellitus are more vulnerable to COVID-19 infection, and are positively associated with worse clinical outcomes [1]. Furthermore, the inconvenience and hesitancy on regular follow-ups resulted by the outbreak of COVID-19 have also produced obstacles to glycemic control in people living with diabetes.

In a national cross sectional study in 2020, the prevalence of diabetes in China was up to 12.8% diagnosed by the 2018 American Diabetes Association (ADA) criteria [2]. The effective vaccines against SARS-CoV-2 are developed in China with great enthusiasm. At the beginning of 2021, health care providers and other vulnerable population in China gradually have received inactivated vaccine against COVID-19. However, among vulnerable individuals, people with poor glycemic control or autoimmune diseases are not recommended for vaccination. Most common adverse reactions after inactivated vaccine in China are injection site pain and fever [3]. To date, no study is conducted to prove the worsening effect of COVID-19 vaccine on glycemic control. While a large proportion of individuals with diabetes are experiencing vaccine hesitancy (VH), which is a main obstacle for the prevalence of vaccination against SARS-CoV-2.

This study, performed on a clinical based sample with regular follow-ups in China, aimed to explore the attitudes of people living with diabetes on COVID-19 vaccination, reasons of the unwillingness to vaccination, and the influence on glycemic control and adverse reactions of vaccines.

## Materials and methods

### Settings and study population

Data were collected from a consecutive series of adults (age > 18 years) with type 2 diabetes under regular follow-ups in the Integrated Care Diabetes Outpatient Clinic of Peking University First Hospital from December 1^st^ to December 31^st^ 2021. All participants provided written informed consent, and the study protocol was approved by the Ethical Board of Peking University First Hospital. People who were incapable of answering online questionnaires were excluded.

### Questionnaire design and data collection

An online questionnaire was conducted, and demographic data including age, sex category, history of drug allergy, history of hypertension and the duration of diabetes was collected. For individuals who hesitated to receive vaccine against COVID-19, main reasons for VH were collected including being afraid of glucose variation, worried about the possibility of leading to other diseases, afraid of allergic reaction, not recommended by physicians, families recommended not to receive vaccination, during pregnancy and other reasons. Fasting blood glucose (FBG), postprandial blood glucose (PBG) and glycosylated hemoglobin A_1c_ (HbA_1c_) measured in the duration of January to March 2021 were collected to match the time of the participants receiving the first and second doses of vaccines. For subjects who have received vaccines against SARS-CoV-2, the date of vaccination, adverse reactions (ARs), recovering time of ARs, and types of inactivated vaccines (CoronaVac® or SinoPharm) were collected. FBG, PBG and HbA_1c_ 1 day to 1 month before and after vaccination were collected from medical records during follow-ups on the purpose of investigating the variation of glycemic control after vaccination. FBG and PBG was the average of three measurements. Most of the participants received their third injection of vaccine on December 2021, thus data of HbA_1c_ after the third dose was not available in our study.

### Statistical analysis

All analysis was performed using SPSS 21.0 (IBM, USA). Student T-tests were performed to assess between-group differences for continuous variables. Chi-square analysis was performed to evaluate differences for categorical variables between groups. Paired T-tests were used for comparing the glucose levels before and after vaccination. Since there was a 28-day time interval between the first and second injection, HbA_1c_ levels at the time point of before vaccination and after the second dose were compared using paired T-tests. A multivariate logistic regression analysis was performed to explore possible confounders for VH, including age, gender, hypertension, history of drug allergy, the duration of diabetes and glucose levels. *P* < 0.05 was considered statistical significant.

## Results

### The characteristics of vaccinated participants and people experienced VH with diabetes

One hundred seventy participants completed the online questionnaire. Among them, 39 (22.9%) subjects experienced VH and had not received vaccine against SARS-CoV-2, and 131 (77.1%) people with diabetes received inactivated vaccine against COVID-19. In people received vaccines, 101 of them (77.1%) received vaccine from CoronaVac®, 30 of them (22.9%) received vaccine from SinoPharm®. Hesitant participants had a higher proportion of female gender (vaccinated group vs. VH group, 62/131 vs. 26/39, *p* = 0.044), higher baseline HbA_1c_ (vaccinated group vs. VH group, 6.56 ± 0.95% vs. 7.54 ± 2.01%, *p* < 0.001) and elevated baseline PBG (vaccinated group vs. VH group, 8.32 ± 1.97 mmol/L vs. 9.44 ± 2.94 mmol/L, *p* = 0.015). The characteristics of the two samples were summarized in Table 1.

**Table 1:** The characteristics of vaccinated participants and people experienced VH with diabetes

	Vaccinated group (*n* = 131)	VH group (*n* = 39)	*p*-value
Gender (male/female)	69/62	13/26	**0.044**
History of Hypertension (n,%)	69, 52.7%	25, 64.1%	0.271
History of drug allergy (n,%)	32, 24.4%	8, 20.5%	0.673
Autoimmune diseases (n,%)	0, 0%	0, 0%	-
Duration of diabetes (year)	8.2 ± 7.4	8.9 ± 7.7	0.585
Baseline types of glucose lowering drugs (n)	2.1 ± 1.2	2.3 ± 1.2	0.281
Baseline HbA_1c_ (%)	6.56 ± 0.95	7.54 ± 2.01	**< 0.001**
Baseline FBG (mmol/L)	6.51 ± 1.04	7.13 ± 2.40	0.053
Baseline PBG (mmol/L)	8.32 ± 1.97	9.44 ± 2.94	**0.015**

*FBG* Fasting blood glucose, *PBG* Postprandial blood glucose, glycosylated hemoglobin A_1c_, HbA_1c_

### Reasons for vaccine hesitancy and risk factors of VH

In 39 participants experienced VH, reasons for hesitating to receive COVID-19 vaccination were as followings: 7 participants (17.9%) feared of glucose variation, 12 participants (30.8%) were worried about the possibility of leading to other diseases, 2 participants (5.1%) were afraid of allergic reaction, 6 participants (15.4%) claimed that COVID-19 vaccination was not recommended by their physicians, 1 participant (2.6%) claimed that his families suggested not to receive vaccination, 10 participants (25.6%) had other reasons, and 1 participants (2.6%) was during pregnancy.

Binary logistic regression analysis was performed to explore factors associated with VH. Subjects of male gender [B = 1.060, odds ratio (OR) = 2.887, *p* = 0.025] and history of hypertension (B = 1.085, OR = 2.959, *p* = 0.021) were likely to get vaccinated, while higher HbA_1c_ was negatively associated with an elevated propensity to receive anti-COVID-19 vaccine (B = -0.573, OR = 0.564, *p* = 0.003). Age, duration of diabetes, history of drug allergy, baseline FBG and PBG did not influence the possibility of VH.

### Adverse reactions after inactivated vaccine against SARS-CoV-2

Adverse events were reported by 40 (30.5%) individuals after the 1^st^ dose of anti-COVID-19 vaccine. Among them, 32 individuals received vaccines of CoronaVac® (32/101, 31.7%) and 8 received vaccines of SinoPharm® (8/30, 26.7%). All adverse events were mild, including myalgia (20/131, 15.3%), followed by fatigue (16/131, 12.2%) and drowsiness (10/131, 7.6%). Headache (2/131, 1.5%), elevated blood pressure (2/131, 1.5%), arthralgia (2/131, 1.5%), fever (1/131, 0.8%) and chills (1/131, 0.8%) were also reported. The median remission time of adverse events was 3 days (0.5-30d).

Adverse reactions after the 2^nd^ injection were reported by 35 (26.7%) individuals, and 23 of them (65.7%) experienced similar adverse events after the first injection. Among them, 25 individuals received vaccines of CoronaVac® (25/101, 24.8%), and 10 individuals received vaccines of SinoPharm® (10/30, 33.3%). Mild adverse events including myalgia (22, 16.8%) were most common, followed by fatigue (8, 6.1%), drowsiness (4, 3.1%) and arthralgia (1, 0.8%).

Of all 131 vaccinated participants, 77 (58.8%) received the 3^rd^ injection. 19 participants (24.7%) reported adverse reactions after the 3^rd^ dose, and 18 received vaccines of CoronaVac® (18/62, 29.0%), 1 received vaccine of SinoPharm® (1/15, 6.7%). Mild adverse reactions of myalgia (12/77, 15.6%), fatigue (5/77, 6.5%) and drowsiness (2/77, 2.6%) were reported.

### Alteration of glycemic control after vaccination

FBG levels fluctuated slightly along with vaccination from 6.51 mmol/L at the baseline to 6.41 mmol/L after the third dose (Table 2). FBG decreased significantly after the third injection compared with FBG after the second dose (second vs. third, 6.78 ± 1.24 vs. 6.41 ± 1.30, *p* = 0.027). PBG levels before and after vaccination did not change significantly from 8.32 mmol/L before vaccination to 7.97 mmol/L after the third injection (Table 2). HbA_1c_ reduced significantly from 6.56% before vaccination to 6.35% after the second injection (*p* = 0.012, Table 2).

**Table 2:** The alteration of glucose levels in vaccinated people living with diabetes

	Baseline (*n* = 131)	After I dose (*n* = 131)	After II dose (*n* = 131)	After III dose (*n* = 77)	*p*-value		
					*p*-value^*^	*p*-value^**^	*p*-value^***^
FBG (mmol/L)	6.51 ± 1.04	6.39 ± 0.98	6.78 ± 1.24	6.41 ± 1.30	0.241	0.802	**0.027**
PBG (mmol/L)	8.32 ± 1.97	7.94 ± 1.61	8.02 ± 1.73	7.97 ± 1.64	0.177	0.523	0.826
HbA_1c_ (%)	6.56 ± 0.95	-	6.35 ± 0.87	-	**0.012**		

*FBG* Fasting blood glucose, *PBG* Postprandial blood glucose, glycosylated hemoglobin A_1c_, HbA_1c_

^*^Baseline vs. after first dose

^**^After first dose vs. after second dose

^***^After second dose vs. after third dose

## Discussion

A global pandemic of COVID-19 was declared from World Health Organization (WHO) on March 11, 2020. People living with diabetes or hyperglycemic state are predisposed to COVID-19 infection. In a single center, retrospective study in patients with COVID-19, hyperglycemia in people with diabetes indicated poor prognosis, as well as secondary hyperglycemia people preceded with COVID-19 infection [4].

Effective vaccines against SARS-CoV-2 are considered a robust prevention from COVID-19 infection. By January 2021, nine vaccines have achieved emergency approval in different countries around the world [5]. In China, healthcare providers and other vulnerable population have received inactivated vaccines (CoronaVac® or SinoPharm®), and population without certified medical contradictions to vaccine are encouraged to receive vaccination. Vaccine hesitancy is commonly seen in individuals with accompanied disease. Since there is a large population of diabetes in China, the safety of vaccines in individuals with diabetes is an inevitable clinic question to be proved. In our single-center study in an outpatient clinic for diabetes, 22.9% participants with diabetes experienced VH, and most common reasons for putting off the vaccination were fearing of causing other diseases (30.8%), followed by fearing of glucose variation (17.9%).

To the best of our knowledge, there is no evidence on the adverse impact on glycemic control of COVID-19 vaccination. A retrospective study in 35 patients with autoimmune diabetes revealed insignificant alteration of glucose level 3 days after the date of vaccine and 14 days preceding the vaccine using continuous glucose monitoring (CGM) [6]. Our study is the first study that proved that the short-term safety of vaccination against COVID-19 on glycemic control. The average FBG remained less than 7 mmol/L before and after vaccine injection, and average PBG remained less than 9 mmol/L. Interestingly, we discovered a significant reduction of HbA_1c_ from 6.56% before vaccination to 6.35% after the second injection (*p* = 0.012), while FBG also decreased significantly after the third injection compared with the second dose (*p* = 0.027). On the contrary to the common sense, vaccines, as an immunological substance that could potentially boost immune response and stress status [7], did not result in poor glucose control. Otherwise, people after vaccine injection were more cautious about their glucose level, which may lead to a glucose lowering effect.

Our conclusion on the impact of vaccines on glycemic control provides new evidence on the safety of vaccines against COVID-19 in the large population with diabetes around the world. However, 14–38% individuals are still hesitant to receive vaccination [8–11]. In a survey on subjects with diabetes in Italy, 18.3% individuals were vaccine hesitant respondents [12], and the rate of VH is lower than that in our study. At multivariate analysis, higher HbA_1c_ and triglycerides were positively correlated with VH in people with type 1 diabetes, while people treated with antihypertensive drugs were more likely to get vaccinated [12]. In people living with type 2 diabetes, obesity and lower levels of creatinine were positively associated with the risk of VH [12]. In our study, people with type 1 diabetes were not enrolled. Similar to previous study, history of hypertension and male subjects with type 2 diabetes had elevated likelihood of vaccination, while higher HbA_1c_ was positively associated with VH. The population of hypertension were male predominant, and approximately 60% people with hypertension were male [13]. However, in our study, after adjustment with gender, history of hypertension was still an independent associating factor of better likelihood of getting vaccinated. Higher HbA_1c_ was another risk factor of VH. In clinical practice, individuals with over-target glycemic control felt hesitated to get vaccinated, and 17.9% people with diabetes declared that they feared of glucose variation among 39 participants experienced VH in our study. Our study innovatively proved the safety of vaccines in glucose alteration, which provided strong evidence on vaccination in people living with diabetes.

In our study, 30.8% participants experienced VH due to fearing of causing other diseases. Adverse reactions of vaccines were mostly mild and transient [14–16]. In a systemic review and meta-analysis, the incidence of pain in individuals administered inactivated vaccines was 31.75%, and the incidences of other systemic adverse reactions were less than 10% [17]. In our study, systemic adverse reactions were reported in 30.5% participants after the first injection of inactivated vaccines, and resolved within 3 days in medium, which was in line with the previous study. People with diabetes should obtain better vaccine confidence with strong evidence on mild adverse reactions and safety on glycemic control.

Some limitations for this study should also be acknowledged. This was a single center study and a relatively small sample size, thus subjects enrolled in this study could not represent the general Chinese population, nor could present the whole population with diabetes in China. In addition, this online survey could only be completed by people with diabetes with higher educational level and media utilization skills, which brought about selective bias to our results.

In conclusions, our study demonstrated that vaccine hesitancy was lower among male subjects and people with hypertension, while vaccine confidence was reduced in people with poor glycemic control. In people living with diabetes, the HbA_1c_ level was lower along with vaccination.

## Data Availability

The raw date of this study was available from the corresponding author on reasonable request.
